# New Orleans Medical and Surgical Journal, Devoted to Medicine and the Collateral Sciences

**Published:** 1845-10

**Authors:** 


NEW ORLEANS MEDICAL AND SURGICAL JOURNAL, DEVOTED TO MEDICINE AND THE COLLATERAL SCIENCES.

We mentioned in the August number of the Examiner, that we had received a circular from New Orleans announcing the intended publication, in that city, of a new Medical Journal, to be edited by Professors Harrison and Carpenter. The last number (Sept.) of the "*New Orleans Medical Journal*" comes to us with its title altered as above, with the names of W. M. Carpenter, M. D., E. D. Fenner M. D., J. Harrison, M. D., and A. Hester, M. D., as Editors. In an editorial signed by them all, we are informed that the interests of the N. O. Medical Journal, and the projected Louisiana Medical and Surgical Journal have been united, and that the Journal will in future appear under the new title, and the joint auspices of the four editors. This concentration of materials and of labor will doubtless be satisfactory to their patrons, whilst it will materially lessen the expense of publication. With so strong a corps of editors, the future numbers of our cotemporary cannot fail to be interesting and instructive.

